# Simulated microgravity enhances CDDP-induced apoptosis signal via p53-independent mechanisms in cancer cells

**DOI:** 10.1371/journal.pone.0219363

**Published:** 2019-07-19

**Authors:** Takahiro Fukazawa, Keiji Tanimoto, Looniva Shrestha, Takeshi Imura, Shinya Takahashi, Taijiro Sueda, Nobuyuki Hirohashi, Eiso Hiyama, Louis Yuge

**Affiliations:** 1 Natural Science Center for Basic Research and Development, Hiroshima University, Hiroshima, Japan; 2 Department of Radiation Disaster Medicine, Research Institute for Radiation Biology and Medicine, Hiroshima University, Hiroshima, Japan; 3 Division of Bio-Environmental Adaptation Sciences, Graduate School of Biomedical and Health Sciences, Hiroshima University, Hiroshima, Japan; 4 Department of Surgery, Graduate School of Biomedical and Health Sciences, Hiroshima University, Hiroshima, Japan; 5 Space Bio-Laboratories Co., Ltd., Hiroshima, Japan; Columbia University, UNITED STATES

## Abstract

Although the biological systems in the human body are affected by the earth’s gravity, information about the underlying molecular mechanisms is limited. For example, apoptotic signaling is enhanced in cancer cells subjected to microgravity. We reasoned that signaling regulated by p53 may be involved because of its role in apoptosis. Therefore, we aimed to clarify the molecular mechanisms of modified cis-diamminedichloroplatinum (CDDP)-sensitivity under simulated microgravity by focusing on p53-related cell death mechanisms. Immunoblotting analyses indicated that, under microgravity, CDDP-induced ATM/p53 signaling increased and caspase-3 was cleaved earlier. However, microgravity decreased the levels of expression of p53 targets *BAX* and *CDKN1A*. Interestingly, microgravity increased the *PTEN*, *DRAM1*, and *PRKAA1* mRNA levels. However, microgravity decreased the levels of mTOR and increased the LC3-II/I ratio, suggesting the activation of autophagy. The CDDP-induced cleavage of caspase-3 was increased during the early phase in Group MG (+), and cleaved caspase-3 was detected even in Group MG (+) with constitutive expression of a mutant type of p53 (hereafter, “+” indicates CDDP treatment). These results interestingly indicate that microgravity altered CDDP sensitivity through activation of caspase-3 by p53-independent mechanism.

## Introduction

Our biological systems work properly in 1G gravity, indicating that those are governed by gravitational force on the earth [1]. The microgravity environment of space flight causes muscle atrophy, decreases bone density, and alters the immune response, as well as other physiological processes [2–5]. Understanding the underlying mechanisms may contribute to the discovery of therapeutic strategies for preventing and treating muscle atrophy and osteoporosis [6,7]. Despite the potential importance of research in this area, research in space is so costly that few researchers are able to conduct the experiments [8,9]. Furthermore, effects of microgravity in space are difficult to distinguish from those of cosmic radiation [10,11].

For these reasons, our group developed the 3D-clinostat to conduct cell culture under simulated microgravity (10^−3^ G). Using the 3D-clinostat, we demonstrated that simulated microgravity affects biological processes such as embryogenesis, stemness of embryonic stem cells, and the differentiation of skeletal muscle [4,12,13]. Furthermore, we found that cis-diamminedichloroplatinum (CDDP) promotes the death of glioma cells cultured under simulated microgravity, and others have found that simulated microgravity promotes the apoptosis of cancer cells [14–17].

The cell death-related mechanisms of apoptosis and autophagy are regulated by p53 signal, at least in part [18–20]. However, little is known about the effects of simulated microgravity on p53-mediated signal transduction. Therefore, here we focused on the importance of simulated microgravity in the regulation of apoptosis signals by p53.

## Materials and methods

### Plasmid construction

Human *TP53* was cloned into the pCMX vector (pCMX-p53) according to a published method [21]. Mutant *TP53* (R248W) was constructed using site-directed mutagenesis of pCMX-p53 (pCMX-p53-R248W). The integrities of the vectors were verified using nucleotide sequence analyses.

### Cell culture

**Fig 1:**
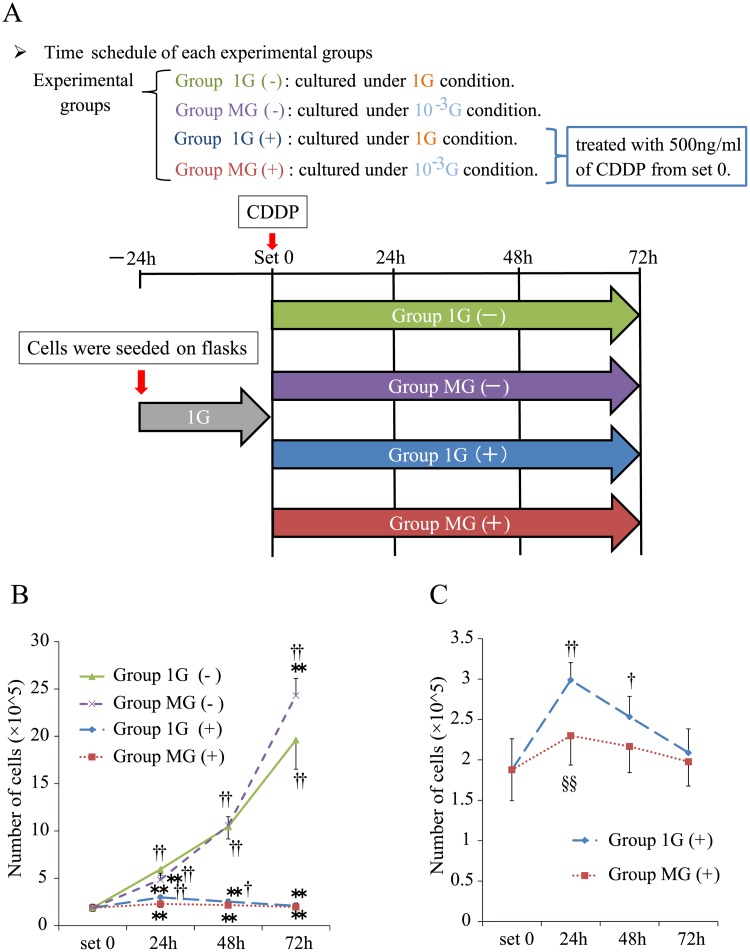
Effect of CDDP on cell growth under normal gravity or simulated microgravity. (A) Time course. HepG2 cells were cultured in 12.5 cm^2^ flasks for 24 h. Then, the flasks were filled with culture medium, divided into the groups described in the Materials and methods section, incubated for 24 h, 48 h, 72 h, and harvested at the indicated times. (B) The number of HepG2 cells in each group was counted at the indicated times. (C) The number of HepG2 cells in Group 1G (+) and Group MG (+). (B and C); Values are represented as the mean ± SD (n = 5); **p < 0.01 vs. Group 1G (−); †p < 0.05 vs. set 0; ††p < 0.01 vs. set 0; §§p < 0.01 vs. Group 1G (+).

The hepatoblastoma cell line, HepG2, was obtained from the Japanese Cancer Research Resource Bank and cultured in RPMI supplemented with 10% fetal bovine serum (FBS), 100 μg/mL kanamycin (Sigma-Aldrich) at 37°C in an atmosphere containing 5% CO_2_. After reaching 80% confluence, the cells were detached using 0.25% trypsin, seeded into a 12.5 cm^2^ flask (2.0 × 10^5^ cells), and cultured for one day. The flasks were subsequently divided into the groups as follows (Fig 1A): HepG2 cells were cultured under normal 1G gravity [Group 1G (−)], simulated microgravity [Group MG (−)], 1G gravity with 500 ng/mL of CDDP [Group 1G (+)], or simulated microgravity with 500 ng/mL of CDDP [Group MG (+)] for 24, 48, or 72 h.

The 3D-clinostat “Gravite” (Space Bio-Laboratories Co., Ltd.) cancels the effect of 1G gravity through controlled rotation of two axes to generate an average of 10^−3^G in 8 min, and the gravity condition was measured by a gravity acceleration sensor. Cells were harvested and counted at the indicated times (Fig 1A). For expression analyses, samples were stored at −80°C. To generate a stably transfected cell line, pCMX without an insert (pCMX-empty) or pCMX-p53-R248W were co-transfected with pDsRed-Monomer-N1 into HepG2 cells treated with TransIT-LT1 Transfection Reagent (Mirus) and then cultured for 2 weeks in growth medium containing 600 μg/mL of G418. Colonies were picked and cultured in T75 flasks in medium containing G418.

### Quantitative real-time RT-PCR

Total RNA was prepared from frozen cell pellets using NucleoSpin RNA (Macherey-Nagel) according to the manufacturer’s protocol. A ReverTra Ace-α Kit (Toyobo) was used to synthesize cDNA. Real-time RT-PCR was performed using a 7500 Real-Time PCR System (Applied Biosystems) and FastStart Universal Probe Master (Roche), following the TaqMan probe method described in manufacturer’s protocol. Standard curves were generated from serial dilutions of cDNA from various cell lines. Gene expression levels were then quantified using 7500 System SDS software version 1.5.1 (Applied Biosystems). *ACTB* (4326315E, Applied Biosystems) was used as an internal control. S1 Table shows other primers and probe sets. Four independent experiments were averaged, and mRNA levels are expressed as a ratio to those of *ACTB* mRNA.

### Immunoblotting

Whole-cell extracts were prepared from frozen cells as described previously [21]. Each protein sample (20 μg) was resolved using 5%–12% gradient sodium dodecyl sulfate-polyacrylamide gel electrophoresis (SDS-PAGE) (ATTO) and blotted onto PVDF membranes (Millipore). To block nonspecific antibody binding, the membranes were incubated with 2% bovine serum albumin in TBS for 1 h at room temperature. Then, membranes were incubated with primary antibodies diluted in CanGet Signal primary buffer (TOYOBO) overnight at 4°C. Primary antibodies and their dilutions were as follows: anti-p53 (OP43, ONCOGENE), 1:1000; anti-phospho-p53 (#9284, Cell Signaling Technology: CST), 1:500; anti-β-actin (A5316, Sigma-Aldrich), 1:5000; anti-ATM (#2873, CST), 1:1000; anti-phospho-ATM (#5883, CST), 1:1000; anti-cleaved Caspase-3 (#9661, CST), 1:500; anti-mTOR (#2983, CST), 1:1000; and anti-LC3A/B (#12741, CST), 1:1000. After washing with TBS-T, the membranes were incubated with horseradish peroxidase (HRP)-linked anti-rabbit IgG (NA934U, GE Healthcare) or anti-mouse IgG (NA931V; GE Healthcare) diluted in CanGet Signal second antibody buffer. After washing, membranes were incubated with the Pierce Femto Western Blotting Substrate, and the immune complexes were detected using X-ray film (GE Healthcare). Experiments were independently performed at least three times. Immunoblots were quantified using ImageJ software (NIH). The expression level of each protein was normalized relative to that of β-actin. To show the autophagy status, the ratio of LC3A/B-II to LC3A/B-I was calculated.

### Statistical analysis

SPSS Statistics version 17.0 (IBM) was used to perform statistical analyses. When compared with set 0, statistical analyses were performed using one-way ANOVA, and Dunnett’s post-hoc test was used to compare the groups. For comparisons of each group analyzed at the same time, the Student’s *t*-test or the Mann–Whitney test was performed as appropriate. P < 0.05 was considered statistically significant.

## Results

### Effects of simulated microgravity on CDDP-induced cell growth inhibition

First, we optimized the conditions for CDDP treatment to analyze its effect on the proliferation or apoptosis signals of HepG2 cells cultured under conditions of normal or simulated microgravity. HepG2 cells were employed because cell death is mediated by wild-type p53. The IC_50_ value of CDDP for HepG2 was determined to be 419.6 ng/mL (Part A in S1 Fig). And immunoblotting demonstrated that 500 ng/mL CDDP was sufficient to activate ATM, p53, and caspase-3 at 48 h (Part B in S1 Fig). Cells were divided into the four groups defined in the Materials and methods section and placed in the 3D-clinostat (Fig 1A). Cells were treated with CDDP and harvested at the indicated times (Fig 1A). CDDP inhibited the proliferation of HepG2 cells (Fig 1B). The numbers of Group MG (−) were higher compared with those of the Group 1G (−) after 72 h (Fig 1B). When cells were treated with CDDP, the number of Group 1G (+) cells increased for 24 h and then started to decline until 72 h. The number of Group MG (+) cells was significantly lower compared with that of Group 1G (+) (Fig 1C).

### Effects of simulated microgravity on apoptosis and autophagy-related signaling

**Fig 2:**
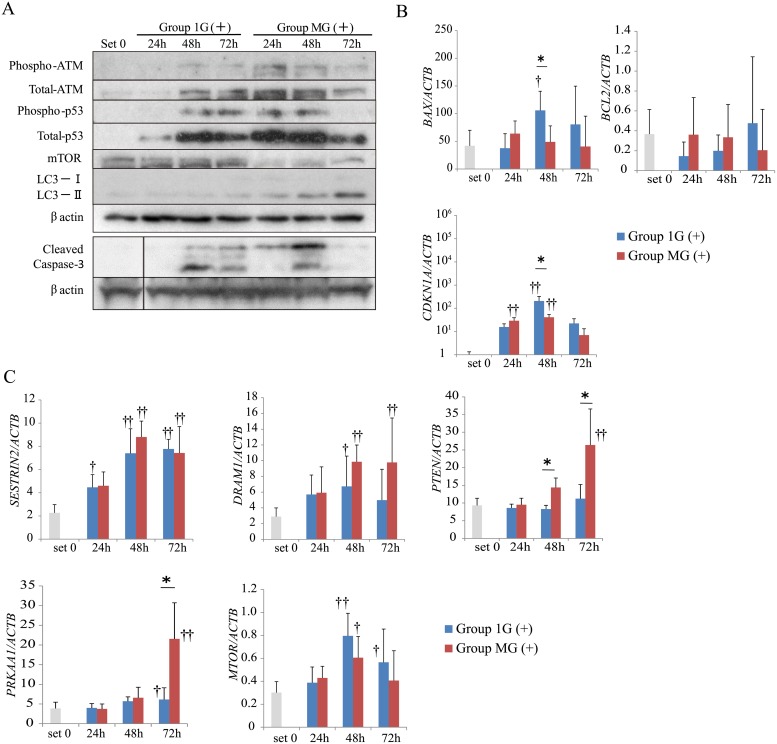
Apoptosis and autophagy-related protein and mRNA levels under normal gravity and simulated microgravity in HepG2 cells treated with CDDP. (A) Immunoblotting was conducted using whole-cell extracts prepared from HepG2 cells of each (Fig 1A). Representative images from more than three independent experiments are shown. (B) Levels of apoptosis-related mRNAs were evaluated using real-time RT-PCR. (C) Levels of autophagy-related mRNAs were evaluated using real-time RT-PCR. (B and C): Relative mRNA levels were calculated as the ratio to *ACTB* levels. The values are expressed as the mean ± SD (n = 4); *p < 0.05; **p < 0.01; †p < 0.05 vs. set 0; ††p < 0.01 vs. set 0.

To clarify the effects of simulated microgravity on apoptosis and autophagy, we used immunoblotting to determine the levels of proteins involved in apoptosis or autophagy. The levels of those were normalized relative to that of β-actin, because the levels of β-actin were not varied between Group 1G (+) and Group MG (+) (Fig 2A). The levels of phosphorylated ATM, total ATM, phosphorylated p53, total p53, and cleaved caspase-3 increased in Group 1G (+), starting after 48 h. In contrast, the levels of Group MG (+) started to increase from 24 h and then decreased after 72 h (Fig 2A and Part A in S3 Fig). The ratio of autophagy-related protein LC3-II/I expressed in Group MG (+) increased in a time-dependent manner. In contrast, the expression of mTOR, whose activation suppresses autophagy signals, decreased in Group MG (+) (Fig 2A and Part A in S3 Fig).

When we determined the levels of mRNAs encoding apoptosis-related proteins, we found that those of the mRNA encoded by the pro-apoptotic gene *BAX* increased after 48 h in Group 1G (+). In contrast, there was no significant change in Group MG (+) (Fig 2B). Furthermore, the levels of the mRNA encoding the anti-apoptotic protein BCL2 were not significantly changed in either group (Fig 2B). The levels of the mRNA encoding the cyclin-dependent kinase inhibitor CDKN1A 48 h after CDDP treatment in Group 1G (+) were significantly higher compared with those of Group MG (+), although they increased after 24 h–48 h and then decreased after 72 h in both groups (Fig 2B). When we determined the levels of mRNAs encoded by autophagy-related genes, we found that those of *SESTRIN2*, *DRAM1*, and *MTOR* increased in both groups. In contrast, the levels of *PTEN* and *PRKAA1* mRNAs increased in Group MG (+) but did not in Group 1G (+) (Fig 2C).

### Effects of CDDP treatment on HepG2 cells transfected with a p53 mutant under simulated microgravity

**Fig 3:**
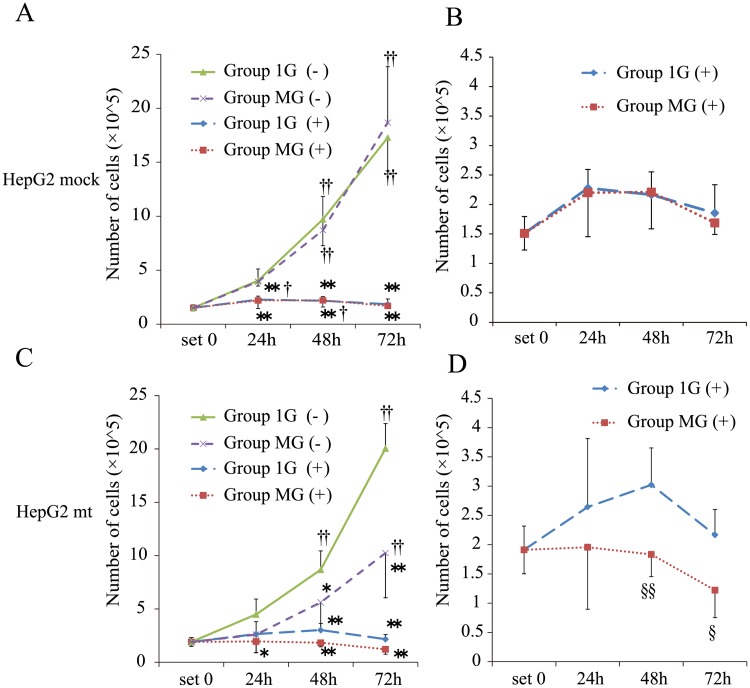
The growth of HepG2 mock cells and HepG2 mt cells under normal gravity or simulated microgravity treated with or without CDDP. (A) The number of HepG2 mock cells in each group was counted following the protocol depicted in Fig 1A. (B) The number of HepG2 mock cells in Group 1G (+) and Group MG (+). (C) The number of HepG2 mt cells in each group. (D) The number of HepG2 mt cells in Group 1G (+) and Group MG (+). (A–D): Values are presented as the mean ± SD (n = 5); *p < 0.05 vs. Group 1G (−); **p < 0.01 vs. Group 1G (−); †p < 0.05 vs. set 0; ††p < 0.01 vs. set 0; §p < 0.05 vs. Group 1G (+); §§p < 0.01 vs. the Group 1G (+).

To determine the role of p53-relating signaling on the cytotoxic effects on Group MG (+), we used HepG2 cells stably transfected with a vector expressing a mutant p53 cDNA, pCMX-p53-R248W (HepG2 mt cells), or cells transfected with the empty pCMX vector (HepG2 mock cells). The dominant-negative effect of enforced expression of the p53 mutant was evaluated using a reporter assay. The p53 target promoter activity was inhibited in pCMX-p53-R248W dose-dependent manner (Part A in S2 Fig). The levels of *BAX* and *CDKN1A* mRNAs in HepG2 mt cells were significantly lower compared with those of HepG2 mock cells (Part B in S2 Fig). HepG2 mt cells proliferated significantly better than HepG2 mock cells (Part C in S2 Fig). Then, HepG2 mock cells or HepG2 mt cells were treated with CDDP under normal or simulated microgravity conditions. CDDP treatment significantly inhibited the proliferation of HepG2 mock cells under both gravitational conditions (Fig 3A and 3B). However, the proliferation of HepG2 mt cells was significantly inhibited under simulated microgravity [Group MG (−)] (Fig 3C). CDDP treatment significantly inhibited the proliferation of HepG2 mt cells [Group MG (+)] compared with those grown under normal gravity [Group 1G (+)] (Fig 3D).

### Effects of the p53 mutant on p53-mediated apoptosis and autophagy-related signaling under simulated microgravity

**Fig 4:**
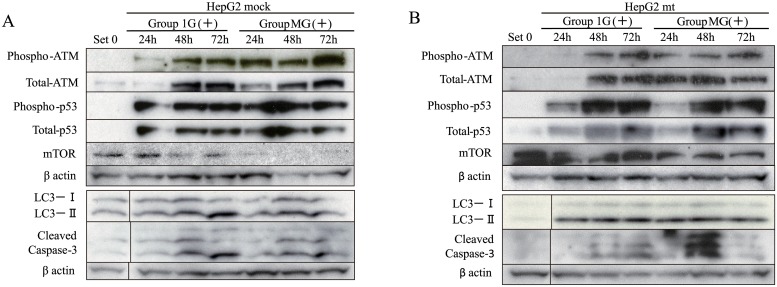
The effect of the p53 mutant on apoptosis and autophagy-related protein levels under normal gravity or simulated microgravity. (A) Immunoblotting was conducted using whole-cell extract prepared from HepG2 mock cells in each group, following the protocol depicted in Fig 1A. Representative images from three independent experiments. (B) Whole-cell extract prepared from HepG2 mt cells was similarly evaluated. Representative images from three independent experiments are shown.

Next, we determined the levels of proteins involved in apoptosis and autophagy. The levels of those were normalized relative to that of β-actin, because the levels of β-actin were not varied between Group 1G (+) and Group MG (+) (Fig 4A and 4B). The levels of phosphorylated ATM, total ATM, phosphorylated p53, and total p53 increased after 48 h in Group 1G (+), whereas they increased after 24 h in Group MG (+) (Fig 4A and Part B in S3 Fig). Compared with native HepG2 cells, ATM/p53 signaling was increased in HepG2 mock cells (Figs 2A and 4A, and Parts A and B in S3 Fig). Expression levels of mTOR in HepG2 mock were suppressed under simulated microgravity [Group MG (+)] same as that in native HepG2 (Figs 2A and 4A, and Parts A and B in S3 Fig). The ratio of LC3-II/I was unchanged in untreated HepG2 mock cells, but increased in native HepG2 cells.

p53-mediated apoptosis and autophagy-related signaling in HepG2 mock cells was not significantly different from that of native HepG2 cells (Figs 2A and 4A, and Parts A and B in S3 Fig).

Then, we evaluated p53-mediated signaling in HepG2 mt cells cultured under normal gravity or microgravity. The kinetics of phosphorylation and total ATM were identical in both cell types under both conditions (Fig 4A and 4B, and Parts B and C in S3 Fig). The levels of total and phosphorylated p53 in HepG2 mt cells gradually increased until 72 h after CDDP treatment under both conditions, and the kinetics were similar to those of HepG2 mock cells. Similar to HepG2 mock cells, the levels of cleaved caspase-3 in HepG2 mt cells increased gradually after CDDP treatment in Group 1G (+). Furthermore, the increase in the levels of cleaved caspase-3 in HepG2 mt cells in Group MG (+) was higher compared with those of HepG2 mock cells. Compared with HepG2 mock cells, the levels of mTOR in HepG2 mt cells were not reduced in Group MG (+) (Fig 4A and 4B, and Parts B and C in S3 Fig). The ratio of LC3-II/I in HepG2 mt cells treated with CDDP was higher than that in HepG2 mock cells; however, significant differences were not observed between gravitational conditions (Fig 4A and 4B, and Parts B and C in S3 Fig).

**Fig 5:**
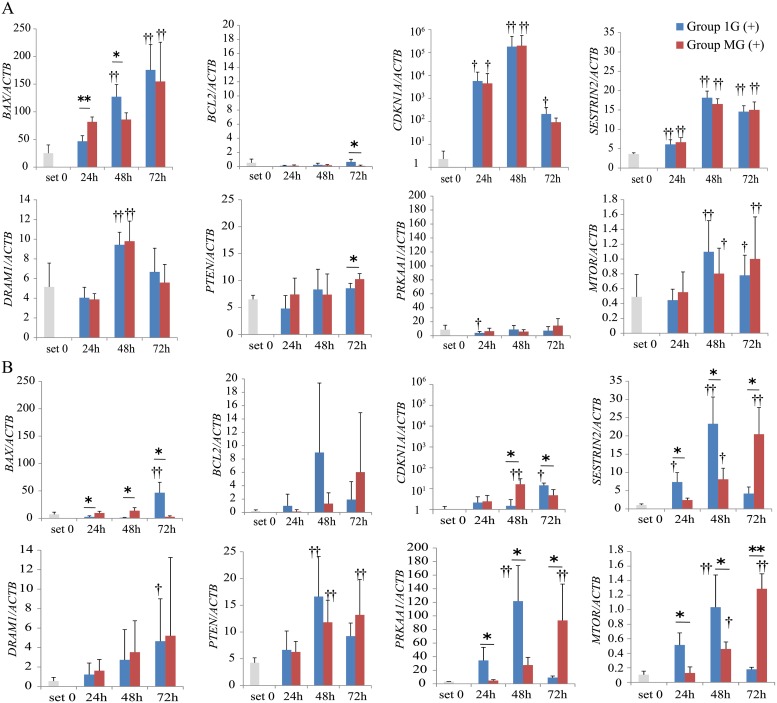
The effect of p53 mutant on apoptosis and autophagy-related gene expressions. (A) Real-time RT-PCR shows the expression levels of apoptosis and autophagy-related genes in HepG2 mock cells. (B) Real-time RT-PCR shows the expression levels of apoptosis and autophagy-related genes in HepG2 mt cells. (A) and (B): relative gene expression levels were calculated as a ratio to *ACTB* expression for each experiment and values are expressed as the mean ± SD (n = 4); *p < 0.05; **p < 0.01; †p < 0.05 vs. set 0; ††p < 0.01 vs. set 0.

Next, we evaluated the levels of mRNAs related to apoptosis and autophagy in HepG2 mock cells. The levels of *BAX* mRNA in HepG2 mock cells increased with CDDP treatment in a time-dependent manner under both gravitational conditions. *BAX* levels were higher in Group MG (+) after 24 h but decreased by 48 h (Fig 5A). The levels of *BCL2* mRNA increased only in Group 1G (+) (Fig 5A). The levels of *CDKN1A*, *SESTRIN2*, *DRAM1*, and *MTOR* mRNAs significantly increased in response to CDDP treatment, but there were no significant differences between conditions (Fig 5A). Any specific changes in expression levels of *PTEN* and *PRKAA1* were found (Fig 5A).

The levels of *BAX* and *CDKN1A* mRNAs in HepG2 mt cells were significantly lower compared with those of HepG2 mock cells (Fig 5A and 5B). The levels of *BAX* and *CDKN1A* mRNAs in HepG2 mt cells increased after 72 h in Group 1G (+), while those in Group MG (+) increased by 48 h (Fig 5B). The levels of *SESTRIN2*, *DRAM1*, *PTEN*, *PRKAA1*, and *MTOR* in HepG2 mt cells increased after CDDP treatment under both gravitational conditions but differed in their kinetics. The levels of *SESTRIN2*, *PTEN*, *PRKAA1*, and *MTOR* in Group 1G (+) increased after 24–48 hours; however, in Group MG (+), they increased after 72 h (Fig 5B).

## Discussion

Although simulated microgravity induces apoptosis in certain cancer cell lines [14–17], the detailed mechanism is unknown. Here we investigated the molecular mechanisms of apoptotic cell death under simulated microgravity, focusing on p53-mediated signaling. We found that HepG2 cells grew better under simulated microgravity. Furthermore, compared with normal gravity, the proliferation of HepG2 cells under simulated microgravity was effectively inhibited after treatment with CDDP. Although the proliferation of native HepG2 cells without or with CDDP treatment differed significantly between normal gravity and simulated microgravity, there was no significant difference when we tested HepG2 mock cells. We speculate that the transfection or G418-selection protocols may have influenced the cell cycle of HepG2 cells. This finding requires further research. HepG2 mt cells grew more than HepG2 mock cells under normal gravity (Parts C in S2 Fig). In contrast, the proliferation of HepG2 mt cells was significantly inhibited under simulated microgravity without or with CDDP treatment. These results suggest that artificial control over gravity or gravity-influenced signal transduction may serve as an anticancer strategy to treat cancer cells that express a mutant p53.

We investigated ATM/p53-mediated apoptotic signaling, since ATM, an upstream regulator of p53, responds to DNA damage caused by CDDP treatment [22]. Specifically, we found that CDDP induced the synthesis of total and phosphorylated forms of ATM and p53 in native HepG2 cells under normal gravity. These surrogates of signaling in HepG2 mock cells and HepG2 mt cells were similarly induced under normal gravity. Under simulated microgravity, the induction of total and phosphorylated forms of ATM and p53 in native HepG2 cells and HepG2 mock cells was enhanced early. In HepG2 mt cells under simulated microgravity, ATM was regulated similarly to native HepG2 cells and HepG2 mock cells. In contrast, differences in gravity did not significantly influence the levels of unphosphorylated and phosphorylated p53.

Cleaved caspase-3 in native HepG2 cells and HepG2 mock cells was induced with CDDP under normal gravity, but cleavage occurred earlier under simulated microgravity, suggesting the acceleration of apoptosis. The levels of cleaved caspase-3 in HepG2 mt cells were lower under normal gravity, although they were increased under simulated microgravity, suggesting the induction of apoptosis. The levels of *BAX* mRNA were significantly higher in HepG2 mt cells under simulated microgravity, although the levels were much lower compared with those of native HepG2 cells or HepG2 mock cells. These results suggest that the effects of CDDP treatment on ATM/p53 signaling may be altered by gravitational changes but we cannot completely explain the inhibition of growth of HepG2 mt cells under simulated microgravity.

p53 signaling plays a key role in apoptosis [18]. Therefore, we investigated the expression of p53 and its upstream and downstream targets. Our results suggest that simulated microgravity enhanced the activity of the ATM/p53 signal transduction pathway early (Fig 2A and Part A in S3 Fig). Moreover, the expression of DNA repair-related genes is down-regulated under simulated microgravity [23], suggesting that the increased activity of ATM at an early phase following drug treatment might be related to the down-regulation of the components of the DNA repair system. Despite the activation of p53 after 24–48 h under simulated microgravity, the levels of the mRNA encoding the p53 target *BAX*, which initiates apoptosis, were not increased (Fig 2B), suggesting that downstream signaling of p53 was inhibited under simulated microgravity. Further investigations will include promoter and ChIP assay, because epigenetic regulation is modulated during space flight [12,24].

Our results using native HepG2 cells or HepG2 mock cells under simulated microgravity show that caspase-3 activity was increased, although p53/BAX signaling was attenuated, suggesting a possible mechanism of activation of caspase-3 induced by a pathway or pathways other than p53/BAX. Moreover, this hypothesis is supported by our observation that caspase-3 was activated in HepG2 mt cells under simulated microgravity (Fig 4A and 4B, and Parts B and C in S3 Fig). Together, our results suggest that ATM/p53/BAX signaling induced by CDDP may be altered by gravitational changes but does not play a crucial role in the activation of caspase-3 under simulated microgravity. Furthermore, caspase-3 is regulated by other signaling pathways, such as FASL/FAS/Caspase-8 pathway and the endoplasmic reticulum stress-induced cell death pathway [25,26]. Further experiments are required to clarify the mechanisms of activation of caspase-3 under simulated microgravity.

We further analyzed intracellular signals relating to autophagy, because autophagy is increased under simulated microgravity [27,28]. Autophagy is regulated by numerous membrane molecules, which form the autophagosome [29]. Among them, LC3-II is generally used as a marker of autophagy [30]. In agreement with previous studies [27,28], we found here that the ratios of LC3-II/I in native HepG2 cells under simulated microgravity, but not under normal gravity, were increased after CDDP treatment. Interestingly, the expression of mTOR whose activation suppresses autophagy, decreased in native HepG2 cells and HepG2 mock cells under simulated microgravity. In contrast, the ratios of LC3-II/I in HepG2 mt cells increased with CDDP treatment under both gravitational conditions, and the levels of mTOR were not significantly different. However, the levels and kinetics of autophagy-related gene products differed between gravitational conditions. These results suggest that CDDP-induced autophagy was activated under simulated microgravity and was enhanced in cells expressing a mutant p53, independent of the strength of the gravitational field.

Autophagy plays a key role in influencing apoptosis and is regulated by the products of the p53-targets *SESTRIN2*, *DRAM1*, *PTEN*, and *PRKAA1* [20,31–33]. Although the levels of *PTEN* and *PRKAA1* mRNAs, whose products are upstream regulators of mTOR, were specifically increased 72 h after CDDP treatment, while the levels of mTOR started to decrease 24 h after CDDP treatment, suggesting the regulation of mTOR activity by p53-independent signaling events. Furthermore, CDDP treatment increased the ratios of LC3-II/I in HepG2 mt cells under both normal and simulated microgravity conditions, independent of the levels of expression of genes that encode upstream components of the autophagy pathway (Figs 4B and 5B, and Part C in S3 Fig). Moreover, the levels of *MTOR* mRNA were unchanged under simulated microgravity, although protein levels were decreased (Fig 2A and 2C, and Part A in S3 Fig). These findings suggest that microgravity affects the posttranslational regulation of the expression of mTOR, including translation, protein stability, or degradation [34,35].

In summary, here we used a 3D-clinostat to detect death-related signals in cells cultured under simulated microgravity. We found that simulated microgravity affected diverse cell deaths-related signals, which were maintained or inhibited, suggesting that normal gravity was important for the transduction, at least in part, of cell death-related signals. Moreover, we show that the proliferation of HepG2 cells expressing a mutant type p53 was inhibited under simulated microgravity without or with CDDP treatment. Further analysis of the pathway may contribute to developing anticancer strategies for cancer cells that express a mutant p53. To our knowledge, we demonstrate for the first time that the activation of caspase-3 induced by CDDP treatment under simulated microgravity was independent of p53 status, suggesting that enhanced apoptosis signal under simulated microgravity is regulated through signals other than those transmitted by p53. Therefore, understanding of the cell death mechanisms that operate under simulated microgravity may contribute to identifying a novel factor that regulates cell death or proliferation. Such a discovery may facilitate the development of a new therapeutic approach for regenerative medicine or cancer therapy.

## Supporting information

S1 Fig The concentration of CDDP was determined by MTT assay and immunoblotting. (A) HepG2 cells were treated with various concentrations of CDDP from 0 to 10000 ng/mL for 72 h. The MTT assay was then performed, and the drug concentration of 50% absorbance of the control was calculated as the IC_50_. (B) HepG2 cells were treated with 500 ng/mL CDDP for 0, 24, 48, or 72 h. Immunoblotting was conducted using whole-cell extracts prepared from HepG2 cells at each time point. Representative images from three independent experiments are shown. (A) The line graphs of percent cell viability relative to control are shown as the mean ± SD; (B) Expression levels for each immunoblot were quantified; relative protein levels were calculated as the ratio to β-actin level. Values are expressed as mean ± SE.
(EPS)

S2 Fig p53 target promoter activities were inhibited by pCMX-p53-R248W. (A) HepG2 cells were transiently transfected with different amounts of the p53 luciferase reporter vectors pCMX-p53 (p53wt) and pCMX-p53-R248W (p53 mt). Promoter activity was calculated as the ratio of firefly to renilla luciferase activities. (B) Real-time RT-PCR analysis of the expression of p53 target genes in HepG2 mock cells and HepG2 mt cells. (C) The number of HepG2 mock cells and HepG2 mt cells cultured for 72 h under normal gravity. (A) Values are expressed as the mean ± SD (n = 5); **p < 0.01, (B) Relative mRNA levels were calculated as the ratio to *ACTB* mRNA levels for each experiment, and the values are expressed as the mean ± SD (n = 3). *p < 0.05; **p < 0.01, (C) Value are represented as the mean ± SD (n = 5); **p < 0.01.
(EPS)

S3 Fig Quantification of immunoblots. (A) The immunoblots in Fig 2A are quantified and their graphs are shown. (B) The immunoblots in Fig 4A are quantified and their graphs are shown. (C) The immunoblots in Fig 4B are quantified and their graphs are shown; (A-C): Relative protein levels were calculated as the ratio to β-actin level. With regard to LC3A/B, the LC3 A/B I to LC3 A/B II ratio was calculated. Values are expressed as mean ± SE.
(EPS)

S4 Fig Summary of the effect of simulated microgravity on p53-mediated signal transduction pathways. (A) The effects of p53-related signaling in Group 1G (+) and Group MG (+). (B) HepG2 mt cells. The effects of p53-mediated signals in Group 1G (+) and Group MG (+).
(EPS)

S1 Table Primer and probe sets for real-time RT-PCR. (DOCX)

S1 Text (DOCX)

## References

[1] Bizzarri M, Monici M, van Loon JJ. How microgravity affects the biology of living systems. Biomed Res Int. 2015;. 10.1155/2015/863075 PMC4312564 25667927

[2] Demontis GC, Germani MM, Caiani EG, Barravecchia I, Passino C, Angeloni D. Human Pathophysiological Adaptations to the Space Environment. Front Physiol. 2017; 8: 547 10.3389/fphys.2017.00547 28824446 PMC5539130

[3] Sibonga JD. Spaceflight-induced bone loss: is there an osteoporosis risk? Curr Osteoporos Rep. 2013; 11: 92–98. 10.1007/s11914-013-0136-5 23564190

[4] Wakayama S, Kawahara Y, Li C, Yamagata K, Yuge L, Wakayama T. Detrimental effects of microgravity on mouse preimplantation development in vitro. PLoS One. 2009; 4: e6753 10.1371/journal.pone.0006753 19707597 PMC2727478

[5] Yi B, Rykova M, Jager G, Feuerecker M, Horl M, Matzel S, et al Influences of large sets of environmental exposures on immune responses in healthy adult men. Sci Rep. 2015; 5: 13367 10.1038/srep13367 26306804 PMC4549790

[6] Cao Q, Zhang J, Liu H, Wu Q, Chen J, Chen GQ. The mechanism of anti-osteoporosis effects of 3-hydroxybutyrate and derivatives under simulated microgravity. Biomaterials. 2014; 35: 8273–8283. 10.1016/j.biomaterials.2014.06.020 24976243

[7] Lee YH, Seo DH, Park JH, Kabayama K, Opitz J, Lee KH, et al Effect of Oenothera odorata Root Extract on Microgravity and Disuse-Induced Muscle Atrophy. Evid Based Complement Alternat Med. 2015;. 10.1155/2015/130513 PMC4405223 25945103

[8] Hand E. Space-station rendezvous set to spur research push. Nature. 2012; 484: 426–427. 10.1038/484426a 22538577

[9] Ratliff D. The next frontier: stem cells and the Center for the Advancement of Science in Space. Stem Cells Dev. 2013; 22: 94–95. 10.1089/scd.2013.0447 24304084

[10] Kiefer J, Pross HD. Space radiation effects and microgravity. Mutat Res. 1999; 430: 299–305. 10.1016/s0027-5107(99)00142-6 10631345

[11] Moreno-Villanueva M, Wong M, Lu T, Zhang Y, Wu H. Interplay of space radiation and microgravity in DNA damage and DNA damage response. NPJ Microgravity. 2017; 3: 14 10.1038/s41526-017-0019-7 28649636 PMC5460239

[12] Furukawa T, Tanimoto K, Fukazawa T, Imura T, Kawahara Y, Yuge L. Simulated microgravity attenuates myogenic differentiation via epigenetic regulations. NPJ Microgravity. 2018; 4: 11 10.1038/s41526-018-0045-0 29845109 PMC5966377

[13] Kawahara Y, Manabe T, Matsumoto M, Kajiume T, Matsumoto M, Yuge L. LIF-free embryonic stem cell culture in simulated microgravity. PLoS One. 2009; 4: e6343 10.1371/journal.pone.0006343 19626124 PMC2710515

[14] Arun RP, Sivanesan D, Vidyasekar P, Verma RS. PTEN/FOXO3/AKT pathway regulates cell death and mediates morphogenetic differentiation of Colorectal Cancer Cells under Simulated Microgravity. Sci Rep. 2017; 7: 5952 10.1038/s41598-017-06416-4 28729699 PMC5519599

[15] Lin SC, Gou GH, Hsia CW, Ho CW, Huang KL, Wu YF, et al Simulated microgravity disrupts cytoskeleton organization and increases apoptosis of rat neural crest stem cells via upregulating CXCR4 expression and RhoA-ROCK1-p38 MAPK-p53 signaling. Stem Cells Dev. 2016; 25: 1172–1193. 10.1089/scd.2016.0040 27269634

[16] Takeda M, Magaki T, Okazaki T, Kawahara Y, Manabe T, Yuge L, et al Effects of simulated microgravity on proliferation and chemosensitivity in malignant glioma cells. Neurosci Lett. 2009; 463: 54–59. 10.1016/j.neulet.2009.07.045 19628020

[17] Zhao J, Ma H, Wu L, Cao L, Yang Q, Dong H, et al The influence of simulated microgravity on proliferation and apoptosis in U251 glioma cells. Vitro Cell Dev Biol Anim. 2017; 53: 744–751. 10.1007/s11626-017-0178-6 28707224

[18] Amaral JD, Xavier JM, Steer CJ, Rodrigues CM. Targeting the p53 pathway of apoptosis. Curr Pharm Des. 2010; 16: 2493–2503. 20500145 10.2174/138161210791959818

[19] Tang J, Di J, Cao H, Bai J, Zheng J. p53-mediated autophagic regulation: A prospective strategy for cancer therapy. Cancer Lett. 2015; 363: 101–107. 10.1016/j.canlet.2015.04.014 25896632

[20] Thorburn A. Apoptosis and autophagy: regulatory connections between two supposedly different processes. Apoptosis. 2008; 13: 1–9. 10.1007/s10495-007-0154-9 17990121 PMC2601595

[21] Tanimoto K, Makino Y, Pereira T, Poellinger L. Mechanism of regulation of the hypoxia-inducible factor-1 alpha by the von Hippel-Lindau tumor suppressor protein. EMBO J. 2000; 19: 4298–4309. 10.1093/emboj/19.16.4298 10944113 PMC302039

[22] Lee JH, Paull TT. Activation and regulation of ATM kinase activity in response to DNA double-strand breaks. Oncogene. 2007; 26: 7741–7748. 10.1038/sj.onc.1210872 18066086

[23] Kumari R, Singh KP, Dumond JW Jr. Simulated microgravity decreases DNA repair capacity and induces DNA damage in human lymphocytes. J Cell Biochem. 2009; 107: 723–731. 10.1002/jcb.22171 19415677

[24] Singh KP, Kumari R, Dumond JW. Simulated microgravity-induced epigenetic changes in human lymphocytes. J Cell Biochem. 2010; 111: 123–129. 10.1002/jcb.22674 20506542

[25] Kruidering M, Evan GI. Caspase-8 in apoptosis: the beginning of “the end”? IUBMB Life. 2000; 50: 85–90. 10.1080/713803693 11185963

[26] Szegezdi E, Fitzgerald U, Samali A. Caspase-12 and ER-stress-mediated apoptosis: the story so far. Ann N Y Acad Sci. 2003; 1010: 186–194. 10.1196/annals.1299.032 15033718

[27] Ryu HW, Choi SH, Namkoong S, Jang IS, Seo DH, Choi I, et al Simulated microgravity contributes to autophagy induction by regulating AMP-activated protein kinase. DNA Cell Biol. 2014; 33: 128–135. 10.1089/dna.2013.2089 24387300

[28] Wang YC, Lu DY, Shi F, Zhang S, Yang CB, Wang B, et al Clinorotation enhances autophagy in vascular endothelial cells. Biochem Cell Biol. 2013; 91: 309–314. 10.1139/bcb-2013-0029 24032680

[29] Glick D, Barth S, Macleod KF. Autophagy: cellular and molecular mechanisms. J Pathol. 2010; 221: 3–12. 10.1002/path.2697 20225336 PMC2990190

[30] Kabeya Y, Mizushima N, Ueno T, Yamamoto A, Kirisako T, Noda T, et al LC3, a mammalian homologue of yeast Apg8p, is localized in autophagosome membranes after processing. EMBO J. 2000; 19: 5720–5728. 10.1093/emboj/19.21.5720 11060023 PMC305793

[31] Feng Z, Hu W, de Stanchina E, Teresky AK, Jin S, Lowe S, et al The regulation of AMPK beta1, TSC2, and PTEN expression by p53: stress, cell and tissue specificity, and the role of these gene products in modulating the IGF-1-AKT-mTOR pathways. Cancer Res. 2007; 67: 3043–3053. 10.1158/0008-5472.CAN-06-4149 17409411

[32] Gump JM, Thorburn A. Autophagy and apoptosis: what is the connection? Trends Cell Biol. 2011; 21: 387–392. 10.1016/j.tcb.2011.03.007 21561772 PMC3539742

[33] Maiuri MC, Malik SA, Morselli E, Kepp O, Criollo A, Mouchel PL, et al Stimulation of autophagy by the p53 target gene Sestrin2. Cell Cycle. 2009; 8: 1571–1576. 10.4161/cc.8.10.8498 19377293

[34] Ikemoto M, Nikawa T, Takeda S, Watanabe C, Kitano T, Baldwin KM, et al Space shuttle flight (STS-90) enhances degradation of rat myosin heavy chain in association with activation of ubiquitin-proteasome pathway. FASEB J. 2001; 15: 1279–1281. 10.1096/fj.00-0629fje 11344113

[35] Nikawa T, Ishidoh K, Hirasaka K, Ishihara I, Ikemoto M, Kano M, et al Skeletal muscle gene expression in space-flown rats. FASEB J. 2004; 18: 522–524. 10.1096/fj.03-0419fje 14715702

