# Supplementary material for: Simulated microgravity enhances CDDP-induced apoptosis signal via p53-independent mechanisms in cancer cells
Source: PLoS One. 2019 Jul 19;14(7):e0219363. doi: 10.1371/journal.pone.0219363 (PMC6641656; doi:10.1371/journal.pone.0219363)
Supplement: S1 Table — (DOCX) [file pone.0219363.s005.docx]

*BAX* 　 Forward: 5′-CCATCATGGGCTGGACAT-3′

Reverse: 5′-CACTCCCGCCACAAAGAT-3′

Universal Probe Library: #69

*BCL2* 　 Forward: 5′-TTGGTATCCTTCTCTTTCACGCAC-3′

Reverse: 5′-ATGGCATTGACGAAGAGGAT-3′

Universal Probe Library: #23

*CDKN1A* Forward: 5′-TGGGCTGCCTGTTTTCAG-3′

Reverse: 5′-AGCTGCTCGCTGTCCACT-3′

Universal Probe Library: #70

*SESTRIN2* Forward: 5′-CGCTTTGAGCTGGAGAAGTC-3′

Reverse: 5′-TCCACAAAGCACAGCATGTC-3′

Universal Probe Library: #1

*DRAM1* Forward: 5′-TGTCTGTGCTTCACTAATTTCCA-3′

Reverse: 5′-TCACAGATCGCACTCACTACG-3′

Universal Probe Library: #78

*PTEN*  　 Forward: 5′-GCTACCTGTTAAAGAATCATCTGGA-3′

Reverse: 5′-CTGGCAGACCACAAACTGAG-3′

Universal Probe Library: #59

*PRkAA1* 　 Forward: 5′-TCTCAGGAGGAGAGCTATTTGATT-3′

Reverse: 5′-GAACAGACGCCGACTTTCTTT-3′

Universal Probe Library: #42

*MTOR* Forward: 5′-GCAGCTGCATGGGGTTTA-3′

Reverse: 5′-CCCGAGGGATCATACAGGT-3′

Universal Probe Library: #56
