# Supplementary material for: Simulated microgravity enhances CDDP-induced apoptosis signal via p53-independent mechanisms in cancer cells
Source: PLoS One. 2019 Jul 19;14(7):e0219363. doi: 10.1371/journal.pone.0219363 (PMC6641656; doi:10.1371/journal.pone.0219363)
Supplement: S1 Text — (DOCX) [file pone.0219363.s006.docx]

**Supplementary Materials and Methods.**

**Luciferase reporter assays**

The luciferase reporter vector was constructed in a previous study (Hirao K et al., Int J Oncol 22:1065-1071, 2003). HepG2 cells were seeded into 24-well plates and cultured for one day. The reporter constructs pCMX-p53 and pCMX-p53-R248W were transiently co-transfected into HepG2 cells using TransIT-LT1 Transfection Reagent (TaKaRa Bio, Inc.). The Renilla luciferase vector (pRL-SV40, Promega) was used to assess transfection efficiency. After treatment for one day, luminescence was observed using Biolumat LB 9505 luminometer (Berthold Co.) with the Dual-Luciferase Reporter Assay System (Promega). Promoter activities are reported as the ratio of firefly: renilla luciferase activities, and the average of five independent experiments was calculated.

**MTT assay**

The cell viability and IC_50_ were determined by MTT assay based on previously reported methods (Noguchi T et al., Clin Cancer Res 10:7100-7107, 2004). Briefly, 5 × 10^3^ cells were seeded in 96-well plates. After 72 h, 0.4% MTT solution and 0.1 M monosodium succinate were added sequentially. After 2 h, the MTT formazan precipitate was dissolved in DMSO. The absorbance at 570 and 650 nm (reference) was measured using an EMax®Endpoint ELISA Microplate Reader (Molecular Devices LLC), and the drug concentration of 50% absorbance of the control was calculated as IC_50_.
